# Association of nonalcoholic fatty liver disease and carotid media‐intima thickness: A systematic review and a meta‐analysis

**DOI:** 10.1002/hsr2.1554

**Published:** 2023-09-10

**Authors:** Manouchehr Khoshbaten, Sepideh H. Maleki, Sara Hadad, Amrit Baral, Ana V. Rocha, Laxmi Poudel, Alireza Abdshah

**Affiliations:** ^1^ Liver and Gastrointestinal Diseases Research Center Tabriz University of Medical Sciences Tabriz Iran; ^2^ Department of Pathology Imam Reza Hospital, Tabriz University of Medical Sciences Tabriz Iran; ^3^ Department of Public Health Sciences Miller School of Medicine, University of Miami Miami Florida USA; ^4^ Nepalgunj Medical College Nepalgunj Banke Nepal; ^5^ School of Medicine Tehran University of Medical Sciences Tehran Iran

**Keywords:** atherosclerosis, cardiovascular diseases, carotid arteries, meta‐analysis, NAFLD

## Abstract

**Introduction:**

The relationship between cardiovascular disorders and nonalcoholic fatty liver disease (NAFLD) has been extensively studied. To better pool this data and make a more definite conclusion, we performed a meta‐analysis to evaluate the association between NAFLD and the thickness of media and intima of carotid artery (CIMT) and cardiovascular disorders.

**Methods:**

We searched PubMed, Ovid, Scopus, ProQuest, Web of Science, and the Cochrane Library, and analyzed the pooled data using R studio and the “metafor” package.

**Results:**

The final analysis included a total of 59 studies with 16,179 cases and 26,120 control individuals. NAFLD was shown to be associated with an increase of 0.1231 mm (20.6%) in carotid artery intima‐media thickness (CIMT) (*p* = 0.002, 95% confidence interval [CI]: 0.0462–0.2000) in individuals with NAFLD. The prevalence of atherosclerotic plaques in the carotid arteries and the occurrence of NAFLD are significantly correlated, according to a meta‐analysis based on 17 distinct studies (*p* = 0.001, 1.28–1.43, 95% CI, odds ratio = 1.356).

**Conclusion:**

Patients with increased CIMT are considerably more likely to have NAFLD. Large prospective investigations are required to corroborate these findings and their prognostic significance, along with the effectiveness of the available interventions.

## Key Points

- Nonalcoholic fatty liver disease (NAFLD) is associated with increased carotid intima media thickness.
- NAFLD is associated with increased prevalence of atherosclerotic plaques in the carotid arteries.

## 1 INTRODUCTION

Nonalcoholic fatty liver disease (NAFLD) is a liver condition marked by an excessive accumulation of fat and is brought on by causes other than alcohol and other particular liver‐damaging variables.^1^ Its prevalence is rising in parallel with obesity and metabolic illness rates.^2^ The prevalence of NAFLD varies by country and ranges from 10% to 24%, while obese patients show a frequency of 57% to 74%. 2.6% of children are also impacted, ranging from 22.5% to 52.8% among obese children.^3^ In Asia, its prevalence has been increased; a 3–20‐fold increase in nations such as Japan in last two decades.^4^ Obesity, diabetes mellitus, hyperlipidemia, and metabolic syndrome are among the main risk factors for NAFLD.^5^ It is believed that NAFLD is likely a liver manifestation of metabolic syndrome, and clinical, epidemiological, and biochemical studies strongly support this hypothesis.^5^ Moreover, recent studies have shown that NAFLD is associated with a variety of classic and non‐classic risk factors for cardiovascular disease disorders. In this case, different epidemiological studies have shown that cardiovascular disease is the primary cause of mortality in NAFLD patients.^6^

One of the subclinical indicators of atherosclerosis is carotid intima‐media thickness (CIMT), which is strongly associated with coronary heart disease.^7^ Numerous studies have been conducted to demonstrate the connection between NAFLD and atherosclerosis, and these studies show that there is a connection between NAFLD and an increase in the intima and media of carotid arteries. Aside from being strongly correlated with obesity and other elements of the metabolic syndrome, increased CIMT, plaque, and impaired endothelial flow‐mediated vasodilatation are additional indicators of subclinical atherosclerosis in nonalcoholic fatty liver patients.^8^, ^9^ A variety of chemicals are also released from fatty and inflamed liver in nonalcoholic fatty liver patients, particularly if they also have nonalcoholic steatohepatitis (NASH), which may have a pathogenic role in accelerating atherosclerosis.^10^ Validation of these findings can turn NAFLD into a prognostic and therapeutical target for cardiovascular disorders. Despite several studies, this association has not yet been investigated in a systematic review approach. As a result, we aimed to conduct a systematic and comprehensive review on the association between NAFLD and cardiovascular disorders.

## 2 METHODS

### 2.1 Search strategy and eligibility criteria

This systematic review was conducted upon studies indexed in the PubMed, Ovid, Scopus, ProQuest, Web of science, and the Cochrane Library until July 2, 2023. Searching strategy to find the most relevant studies was (“hepatic steatosis” or “non‐alcoholic fatty liver disease” or “fatty liver”) and (“atherosclerosis” or “intima and media thickness” or “carotid artery” or “carotid plaque” or “cardiovascular disease”). In addition, our detailed search strategy is summarized in Table 1. The inclusion criteria were English‐language descriptive and cohort studies examining the association between adult patients with NAFLD and carotid artery atherosclerosis (over 18 years old). Moreover, studies that have not yet been published, or did not report the variables considered in this study as well as inclusion of patients with hepatic steatosis due to secondary causes (including alcoholic patients, intravenous nutrition, hepatitis B or C, or drugs) were excluded from our repertoire. Our Patient Intervention Comparator Outcome (PICO) was as follows: (1) P: patients with NAFLD, (2) I: ultrasound examination of the carotid artery, (3) C: normal control individuals, and (4) carotid‐intima thickness and its plaque amount. Two separate reviewers screened all the studies, and another investigator entered all the endpoints and measures obtained in the chosen studies in Excel. The data was then prepared and analyzed as explained in the statistical analysis section. No automation tool was used in this process.

**Table 1:** Search strategy in PubMed database and keywords used for search.

Search	Query	Items found
**#1**	**Search: “Atherosclerosis”[Mesh] Sort by: Most Recent**	56,032
**#2**	**Search: ((Atherosclerosis[Title/Abstract]) OR (Atheroscleroses[Title/Abstract])) OR (Atherogenesis[Title/Abstract])**	142,274
**#3**	**Search: (“Atherosclerosis”[Mesh]) OR (((Atherosclerosis[Title/Abstract]) OR (Atheroscleroses[Title/Abstract])) OR (Atherogenesis[Title/Abstract]))**	163,244
**#4**	**Search: “Carotid Arteries”[Mesh] Sort by: Most Recent**	62,693
**#5**	**Search: (“Carotid Artery”[Title/Abstract]) OR (“Carotid Arteries”[Title/Abstract])**	79,303
**#6**	**Search: (“Carotid Arteries”[Mesh]) OR ((“Carotid Artery”[Title/Abstract]) OR (“Carotid Arteries”[Title/Abstract]))**	**108,219**
**#7**	**Search: ((“Atherosclerosis”[Mesh]) OR (((Atherosclerosis[Title/Abstract]) OR (Atheroscleroses[Title/Abstract])) OR (Atherogenesis[Title/Abstract]))) AND ((“Carotid Arteries”[Mesh]) OR ((“Carotid Artery”[Title/Abstract]) OR (“Carotid Arteries”[Title/Abstract])))**	**13,290**
**#8**	**Search: “Carotid Artery Diseases”[Mesh]**	52,225
**#9**	Search: ((((((“Carotid Atherosclerosis”[Title/Abstract]) OR (“Carotid Atheroscleroses”[Title/Abstract])) OR (“Carotid Artery Disease*“[Title/Abstract])) OR (“Carotid Artery Disorder*“[Title/Abstract])) OR (“Carotid Arterial Disease*“[Title/Abstract])) OR (“Carotid artery atherosclerosis”[Title/Abstract])) OR (“Carotid Atherosclerotic Disease*“[Title/Abstract])	8489
**#10**	Search: (“Carotid Artery Diseases”[Mesh]) OR (((((((“Carotid Atherosclerosis”[Title/Abstract]) OR (“Carotid Atheroscleroses”[Title/Abstract])) OR (“Carotid Artery Disease*“[Title/Abstract])) OR (“Carotid Artery Disorder*“[Title/Abstract])) OR (“Carotid Arterial Disease*“[Title/Abstract])) OR (“Carotid artery atherosclerosis”[Title/Abstract])) OR (“Carotid Atherosclerotic Disease*“[Title/Abstract]))	54,602
**#11**	Search: ((“Carotid Artery Diseases”[Mesh]) OR (((((((“Carotid Atherosclerosis”[Title/Abstract]) OR (“Carotid Atheroscleroses”[Title/Abstract])) OR (“Carotid Artery Disease*“[Title/Abstract])) OR (“Carotid Artery Disorder*“[Title/Abstract])) OR (“Carotid Arterial Disease*“[Title/Abstract])) OR (“Carotid artery atherosclerosis”[Title/Abstract])) OR (“Carotid Atherosclerotic Disease*“[Title/Abstract]))) OR (((“Atherosclerosis”[Mesh]) OR (((Atherosclerosis[Title/Abstract]) OR (Atheroscleroses[Title/Abstract])) OR (Atherogenesis[Title/Abstract]))) AND ((“Carotid Arteries”[Mesh]) OR ((“Carotid Artery”[Title/Abstract]) OR (“Carotid Arteries”[Title/Abstract]))))	**61,111**
**#12**	**Search: “Non‐alcoholic Fatty Liver Disease”[Mesh]**	23,321
**#13**	Search: ((((((NAFLD[Title/Abstract]) OR (“Non‐alcoholic Fatty Liver*“[Title/Abstract])) OR (“Nonalcoholic Steatohepatitis”[Title/Abstract])) OR (“Nonalcoholic Steatohepatitides”[Title/Abstract])) OR (“Nonalcoholic Fatty Liver Disease*“[Title/Abstract])) OR (“Non‐alcoholic Fatty Liver Disease*“[Title/Abstract])) OR (“Non alcoholic Fatty Liver Disease*“[Title/Abstract])	**36,568**
**#14**	Search: (“Nonalcoholic Fatty Liver Disease”[Mesh]) OR (((((((NAFLD[Title/Abstract]) OR (“Nonalcoholic Fatty Liver*“[Title/Abstract])) OR (“Nonalcoholic Steatohepatitis”[Title/Abstract])) OR (“Nonalcoholic Steatohepatitides”[Title/Abstract])) OR (“Non‐alcoholic Fatty Liver Disease*“[Title/Abstract])) OR (“Non‐alcoholic Fatty Liver Disease*“[Title/Abstract])) OR (“Non alcoholic Fatty Liver Disease*“[Title/Abstract]))	39,599
**#15**	Search: ((“Non‐alcoholic Fatty Liver Disease”[Mesh]) OR (((((((NAFLD[Title/Abstract]) OR (“Nonalcoholic Fatty Liver*“[Title/Abstract])) OR (“Nonalcoholic Steatohepatitis”[Title/Abstract])) OR (“Nonalcoholic Steatohepatitides”[Title/Abstract])) OR (“Nonalcoholic Fatty Liver Disease*“[Title/Abstract])) OR (“Non‐alcoholic Fatty Liver Disease*“[Title/Abstract])) OR (“Non‐alcoholic Fatty Liver Disease*“[Title/Abstract]))) AND (((“Carotid Artery Diseases”[Mesh]) OR (((((((“Carotid Atherosclerosis”[Title/Abstract]) OR (“Carotid Atheroscleroses”[Title/Abstract])) OR (“Carotid Artery Disease*“[Title/Abstract])) OR (“Carotid Artery Disorder*“[Title/Abstract])) OR (“Carotid Arterial Disease*“[Title/Abstract])) OR (“Carotid artery atherosclerosis”[Title/Abstract])) OR (“Carotid Atherosclerotic Disease*“[Title/Abstract]))) OR (((“Atherosclerosis”[Mesh]) OR (((Atherosclerosis[Title/Abstract]) OR (Atheroscleroses[Title/Abstract])) OR (Atherogenesis[Title/Abstract]))) AND ((“Carotid Arteries”[Mesh]) OR ((“Carotid Artery”[Title/Abstract]) OR (“Carotid Arteries”[Title/Abstract])))))	184
**#16**	**Search: “Carotid Stenosis”[Mesh]**	18,014
**#17**	Search: (((((“Carotid Stenosis”[Title/Abstract]) OR (“Carotid plaques”[Title/Abstract])) OR (“Carotid Stenoses”[Title/Abstract])) OR (“Carotid Ulcer*“[Title/Abstract])) OR (“Intima media thickness”[Title/Abstract])) OR (“Carotid intimal thickness”[Title/Abstract])	22,959
**#18**	Search: (“Carotid Stenosis”[Mesh]) OR ((((((“Carotid Stenosis”[Title/Abstract]) OR (“Carotid plaques”[Title/Abstract])) OR (“Carotid Stenoses”[Title/Abstract])) OR (“Carotid Ulcer*“[Title/Abstract])) OR (“Intima media thickness”[Title/Abstract])) OR (“Carotid intimal thickness”[Title/Abstract]))	35,063
**#19**	Search: ((“Non‐alcoholic Fatty Liver Disease”[Mesh]) OR (((((((NAFLD[Title/Abstract]) OR (“Nonalcoholic Fatty Liver*“[Title/Abstract])) OR (“Nonalcoholic Steatohepatitis”[Title/Abstract])) OR (“Nonalcoholic Steatohepatitides”[Title/Abstract])) OR (“Nonalcoholic Fatty Liver Disease*“[Title/Abstract])) OR (“Non‐alcoholic Fatty Liver Disease*“[Title/Abstract])) OR (“Non alcoholic Fatty Liver Disease*“[Title/Abstract]))) AND ((“Carotid Stenosis”[Mesh]) OR ((((((“Carotid Stenosis”[Title/Abstract]) OR (“Carotid plaques”[Title/Abstract])) OR (“Carotid Stenoses”[Title/Abstract])) OR (“Carotid Ulcer*“[Title/Abstract])) OR (“Intima media thickness”[Title/Abstract])) OR (“Carotid intimal thickness”[Title/Abstract])))	268
**#20**	**Search: “Cerebrovascular Disorders”[Mesh]**	427,170
**#21**	Search: ((((((((“Cardiovascular risk marker*“[Title/Abstract]) OR (“Surrogate markers of cardiovascular disease*“[Title/Abstract])) OR (“Cerebrovascular Disorder*“[Title/Abstract])) OR (“Intracranial Vascular Disease*“[Title/Abstract])) OR (“Intracranial Vascular Disorder*“[Title/Abstract])) OR (“Cerebrovascular Disease*“[Title/Abstract])) OR (“Brain Vascular Disorder*“[Title/Abstract])) OR (“Cerebrovascular Occlusion*“[Title/Abstract])) OR (“Cerebrovascular Insufficienc*“[Title/Abstract])	32,706
**#22**	Search: (“Cerebrovascular Disorders”[Mesh]) OR (((((((((“Cardiovascular risk marker*“[Title/Abstract]) OR (“Surrogate markers of cardiovascular disease*“[Title/Abstract])) OR (“Cerebrovascular Disorder*“[Title/Abstract])) OR (“Intracranial Vascular Disease*“[Title/Abstract])) OR (“Intracranial Vascular Disorder*“[Title/Abstract])) OR (“Cerebrovascular Disease*“[Title/Abstract])) OR (“Brain Vascular Disorder*“[Title/Abstract])) OR (“Cerebrovascular Occlusion*“[Title/Abstract])) OR (“Cerebrovascular Insufficienc*“[Title/Abstract]))	441,976
**#23**	Search: ((“Non‐alcoholic Fatty Liver Disease”[Mesh]) OR (((((((NAFLD[Title/Abstract]) OR (“Nonalcoholic Fatty Liver*“[Title/Abstract])) OR (“Nonalcoholic Steatohepatitis”[Title/Abstract])) OR (“Nonalcoholic Steatohepatitides”[Title/Abstract])) OR (“Nonalcoholic Fatty Liver Disease*“[Title/Abstract])) OR (“Non‐alcoholic Fatty Liver Disease*“[Title/Abstract])) OR (“Non alcoholic Fatty Liver Disease*“[Title/Abstract]))) AND ((“Cerebrovascular Disorders”[Mesh]) OR (((((((((“Cardiovascular risk marker*“[Title/Abstract]) OR (“Surrogate markers of cardiovascular disease*“[Title/Abstract])) OR (“Cerebrovascular Disorder*“[Title/Abstract])) OR (“Intracranial Vascular Disease*“[Title/Abstract])) OR (“Intracranial Vascular Disorder*“[Title/Abstract])) OR (“Cerebrovascular Disease*“[Title/Abstract])) OR (“Brain Vascular Disorder*“[Title/Abstract])) OR (“Cerebrovascular Occlusion*“[Title/Abstract])) OR (“Cerebrovascular Insufficienc*“[Title/Abstract])))	220

### 2.2 Ethical considerations

The information about the patients was kept confidential since this research was done as a review. Only studies that addressed ethical concerns and were published in credible journals were included in this analysis. The findings of this research were disclosed without hindrance, in accordance with the confidentially principle, and by citing the relevant sources. The investigation was carried out with the greatest care to ensure that the conclusions are supported by the available scientific data. To prevent plagiarism, the scientific writing rules were also followed.

### 2.3 Statistical analysis

We have listed the studies that we utilized in along with their details in Table 2. After importing the data, and defining the effects as “the difference between carotid intima‐media thickness of subjects with NAFLD versus those without NAFLD; we performed the analysis using R.^79^ The preparation was done using the “tidyverse”^80^ package, and the meta‐analysis and visualizations were done using the “metafor”^81^ package. For the meta‐analysis, we used the random‐effects model, and we checked for heterogeneity and ran sensitivity analysis. We performed the meta‐analysis both with the overall effect and by using sample number of the studies as weights. We then generated the forest plots and funnel plot to visualize the effects.

**Table 2:** Summary of data obtained from articles.

Carotid atherosclerosis plaque				CIMT(mm)				Number of NAFLD	Number of control patients	Total sample	NAFLD diagnosis	Published year	First author
NAFLD		CONTROL		NAFLD		CONTROL							
Count	%	Count	%	Mean	SD	Mean	SD						
				0.46	0.04	0.41	0.03	320	687	1007	Sonography	2017	Xia Li^11^
	35		31	0.78	0.15	0.75	0.15	636	484	898	Sonography	2020	Hyeok‐Hee Lee^12^
7	14.2	0	0	0.9 (no SD reported)		0.6 (no SD reported)		50	30	80	Biopsy	2014	Nicoleta V. Leach^13^
				32.0% (high)		22.10% (high)		290	290	580	Sonography	2013	Kamran B. Lankarani^14^
				0.4	0.19	0.27	0.18	117	44	161	Sonography	2013	Metin Kucukazman^15^
				0.818	0.006	0.818	0.008	747	464	1211	Sonography	2014	Soo‐Kyung Kim^16^
				0.75	0.06	0.74	0.08	180	1105	1285	CT	2014	Nan Hee Kim^17^
				0.72	0.06	0.619	0.04	103	50	153	Sonography	2019	Cemal Kemaloglu^18^
				0.68	0.1	0.65	0.1	40	26	66	Sonography	2009	F. Karakurt^19^
109	34.1	59	18.8	0.79	0.18	0.73	0.13	320	313	633	Sonography	2012	Ji Hoon Kang^20^
				0.09	0.19	0.8	0.1	29	22	51	CT	2013	Pikkel Josef^21^
357	13.8	865	14.3	0.59	0.1	0.57	0.1	2590	6042	8632	Sonography	2012	Yun Huang^22^
				0.66	0.04	0.64	0.04	342	613	955	Sonography	2016	Ho Cheol Hong^23^
				1.09	0.15	0.88	0.05	196	100	296	Sonography	2018	Amr Shaaban Hanafy^24^
2452	56.5	1883	44.5	0.82	0.3	0.85	0.39	4349	4231	8571	Sonography	2017	Kaifeng Guo^25^
				0.83	0.21	0.76	0.14	106	909	1015	CT	2018	Anders Gummesson^26^
				0.6	0.11	0.54	0.08	113	57	170	Biopsy	2013	Halil Genc^27^
46	50	95	52	0.96	0.22	0.79	0.18	91	182	273	Sonography/biopsy	2016	Anna Ludovica Fracanzani^28^
				0.89	0.26	0.64	0.14	125	350	375	sonography/biopsy	2008	Anna Ludovica Fracanzani^29^
				0.83	0.16	0.77	0.016	49	33	82	Sonography	2018	Reza Fadaei^30^
				0.6	0.13	0.5	0.08	67	35	102	Biopsy	2012	Teoman Dogru^31^
				0·60		0·50		115	74	189	Sonography/biopsy	2013	Teoman Dogru^32^
				0.67	0.09	0.52	0.11	51	21	72	biopsy	2012	Yasar Colaka^33^
				0.68	0.15	0.68	0.14	93	37	130	Sonography/biopsy	2017	Ibrahim Cetindaglı^34^
20	50	10	25	0.70	0.2	0.54	0.13	40	40	80	Sonography	2005	Angel Brea^35^
				0.64	0.17	0.43	0.14	34	35	69	Sonography	2013	Ö. Başar^36^
				0.1	0.02	0.08	0.02	50	50	100	Sonography	2021	Maha Assem^37^
				0.64	0.1	0.52	0.10	57	30	87	Sonography/biopsy	2012	Yasar Colak^38^
9	92.52	58	52.73					110	107	217	Sonography	2015	Florin Casoinic^39^
				0.646	0.091	0.544	0.067	40	40	80	Biopsy	2007	Cem Aygun^40^
60	30.4	70	35.2	0.6	0	0.6	0	148	129	277	Sonography	2017	Clarence Gill^41^
	31		24	0.79	0.22	0.73	0.15	452	453	905	Sonography	2012	Xiaoming Li^42^
				0.81	0.14	0.58	0.15	250	85	335	Sonography	2011	Afshin Mohammadi^43^
				0.65	0.09	0.55	0.07	84	65	149	Sonography	2011	Afshin Mohammadi^44^
38	25.3	8	5.3	0.62	0.19	0.5	0.13	150	150	300	Sonography	2019	Ali Mohammadzadeh^45^
				0.56	0.11	0.45	0.13	99	52	151	Sonography	2014	Maryam Zaare Nahandi^46^
				0.44	0.07	0.4	0.05	61	41	102	Biopsy	2015	Kadir Ozturk^47^
				0.63	0.17	0.54	0.1	45	45	90	Sonography	2020	Vijay Rampally^48^
				0.79	0.18	0.67	0.13	23	28	51	Biopsy	2010	Charalambos Vlachopoulos^49^
				1.14	0.20	0.82	0.12	85	160	245	Biopsy	2006	Giovanni Targher^50^
				0.8	0.1	0.6	0.03	109	109	218	Sonography	2016	Hafsa Riaz^51^
				0.82	0.15	0.58	0.1	200	100	300	Sonography	2017	Abid Rasool^52^
				1.10	0.20	0.84	0.13	50	40	90	Biopsy	2005	Giovanni Targher^53^
				0.6	0.11	0.6	0.23	654	2770	3433	Sonography	2019	Zhuojun Xin^54^
				0.6	0.12	0.49	0.1	40	40	80	Sonography	2012	Manik Lal Thakur^55^
				1.2	0.14	0.9	0.12	37	35	72	Sonography	2016	Radojica V. Stolic^56^
	29.3		33.6	0.82	0.16	0.76	0.15	92	128	220	Sonography	2010	Paolo Salvi^57^
				0.88	0.18	0.87	0.2	729	3394	4123	Sonography	2019	Ebenezer Oni^58^
				0.71	0.17	0.69	0.31	107	43	150	Sonography	2020	Nurazam omar^59^
	19.2		2.2	0.592	0.1	0.489	0.13	101	544	645	Sonography	2013	Sandhya Mishra^60^
	57.8		37.5	0.84	0.1	0.72	0.1	90	64	154	Sonography	2009	Stefano Ramilli^61^
				34.50% (high)		19.10% (high)		144	107	251	Sonography	2018	Eugene Choon‐Li Tan^62^
				1.18	0.14	0.94	0.12	45	40	85	Sonography	2004	Giovanni Targher^63^
				1.09	0.77	0.98	0.68	38	18	56	Sonography	2011	Laura I. Poanta^64^
	31.6		40.1	0.82	0.2	0.64	0.14	73	51	124	Sonography	2018	Sivabal Vanjiappan^65^
27	71.1	4	8	1.1	0.1	0.8	0.1	38	50	88	CT	2014	Ivana Mikolasevic^66^
				0.75	0.23	0.66	0.15	24	28	52	Biopsy	2012	Binnur Pinarbasi^67^
				0.67	0.15	0.63	0.13	394	421	815	Sonography	2015	Seung Hwan Moon^68^
				0.75	0.15	0.74	0.13	61	40	101	CT	2009	Jean Michel Petit^69^
				1	0.6	0.98	0.11	289	47	336	Sonography	2015	Cristina Alina Silaghi^70^
	20.4		0	0.75	0.15	0.58	0.09	77	15	92	Biopsy	2015	Josep Puig^71^
	21.5		6	0.72	0.15	0.46	0.13	117	32	149	Biopsy	2015	Josep Puig^71^
645	40	967	59.9	43.84% (high)		56.15% (high)		1571	2541	4112	Sonography	2018	Jilin Zheng^72^
	28.9		16.9	0.81	0.25	0.69	0.18	123	599	722	Sonography	2016	Lie Zhang^73^
	21.9		15	30% (high)		21.1% (high)		1375	1237	2612	Sonography	2018	Yu Chen Guo^74^
				0.712	0.150	0.5875	0.088	76	24	100	Sonography	2022	Shabbirhussain^75^
				0.78	0.145	0.75	0.15	456	396	852	Sonography	2023	Cho^76^
				0.64	0.1	0.8	0.2	63	35	98	Sonography	2023	Zhang^77^
				31.8% high		19.7% high		384	506	890	Sonography	2022	Bessho^78^

Abbreviations: CT, computed tomography; NAFLD, nonalcoholic fatty liver disease; SD, standard deviation.

## 3 RESULTS

### 3.1 Data collection

**Figure 1:**
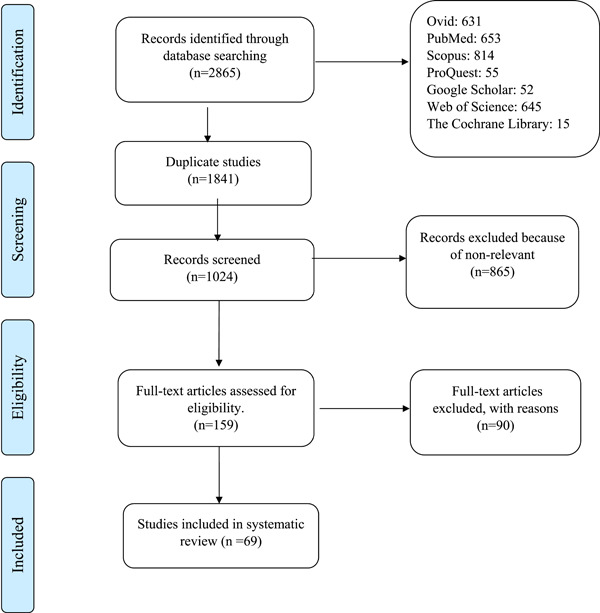
PRISMA flow diagram showing the selection process of studies in the systematic review and meta‐analysis.

PubMed, Ovid, Scopus, ProQuest, Web of Science, and the Cochrane Library databases were used in our study in which 495, 546, 566, 80, 683, and 4 articles were screened out, respectively. Strategy search for PubMed database is provided in Table 1. Consequently, a total of 2865 articles were screened out in which 1024 articles remained after eliminating duplicates from the total articles, while 159 items remained after initial screening using titles and abstracts. The whole texts of 159 articles were meticulously examined in the next stage. Then, 90 items were eliminated as being irrelevant and the data from 69 remained articles was collected, which is summarized in Table 2. There was a total of 56,582 patients in these studies, comprising 26,689 NAFLD cases and 38,584 control subjects. Finally, 10 articles were excluded due to reporting mean and percentage values rather than mean and standard deviation, and 59 articles remained for meta‐analysis. The details of included and excluded data can be shown in illustrated PRISMA flowchart (Figure 1).

### 3.2 Relationship between NAFLD and CIMT

**Figure 2:**
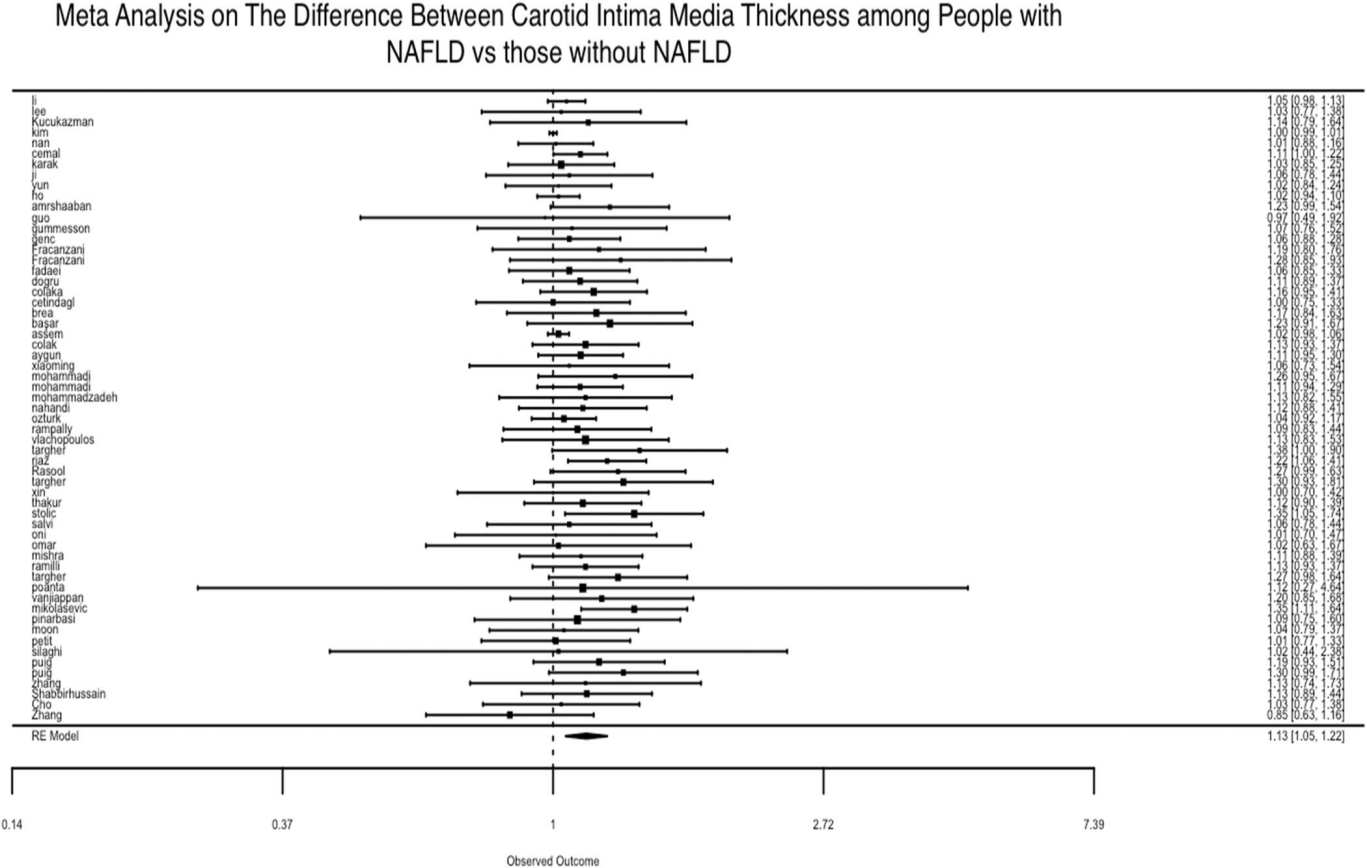
Results of a meta‐analysis of studies on the association of carotid artery intima‐media diameter (CIMT) with nonalcoholic fatty liver disease (NAFLD).

**Figure 3:**
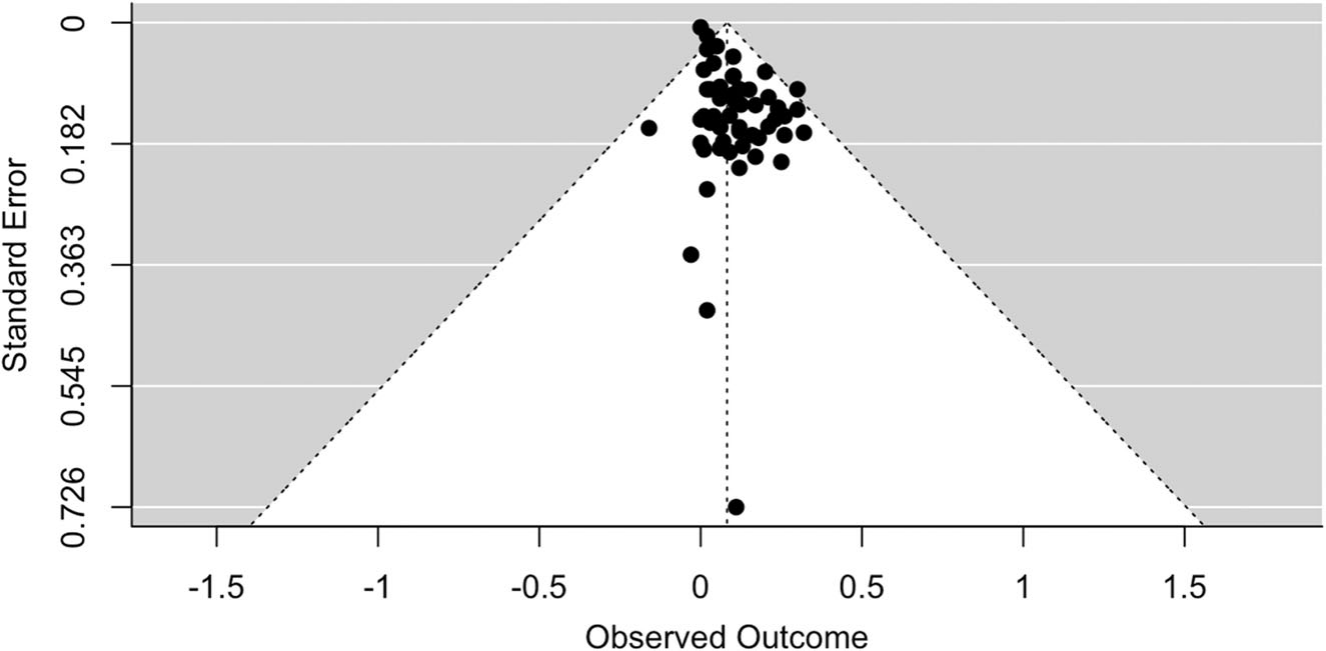
Funnel plot publication bias in the studies conducted on the relationship between carotid artery intima‐media diameter (CIMT) and nonalcoholic fatty liver disease (NAFLD).

The findings of the meta‐analysis based on 59 studies (after weighing based on their sample size) revealed that NAFLD was linked to an increase in CIMT associated with an increase of **0.1231 mm** (20.6%) in CIMT (*p* = 0.002, 95% CI: **0.0462, 0.2000**) in individuals with NAFLD. The forest plot in Figure 2 shows our pooled estimate, and the funnel plot in Figure 3 shows that the studies were symmetrically distributed.

At first we noted a significant heterogeneity among the studies, but after further exploration, we noted that one of the studies was causing a significant heterogeneity, and because of the lower sample size and the wide confidence interval in the study,^21^ we excluded the study from the meta‐analysis. In the final analysis, using the sample size of the studies as weights, we noted no significant heterogeneity among the studies (Q = 67.49, and *p* = 0.18).

**Figure 4:**
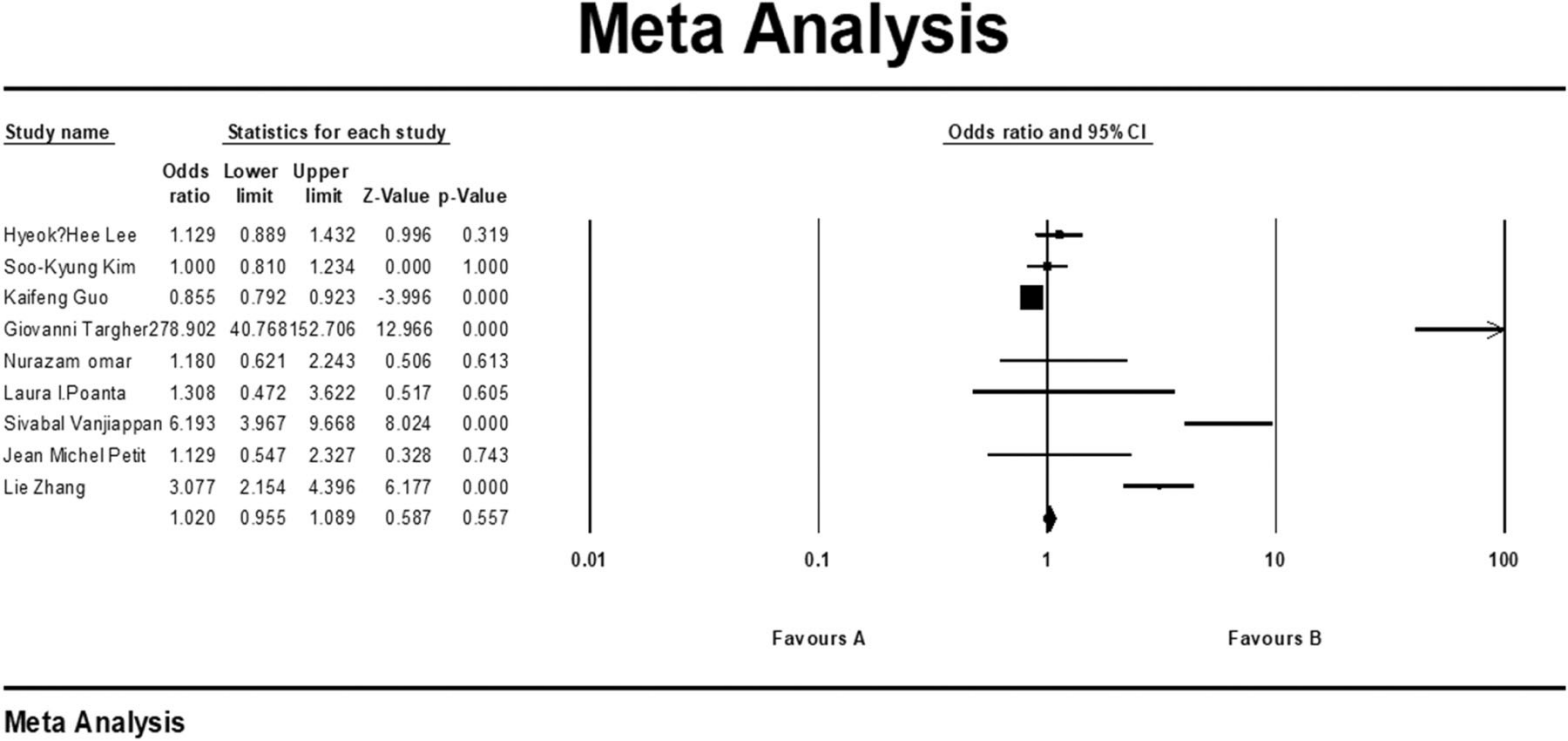
Subgroup results of meta‐analysis of studies conducted on diabetic patients regarding the relationship between carotid artery intima‐media diameter (CIMT) and nonalcoholic fatty liver disease (NAFLD). CI, confidence interval.

Also, we looked in several studies on patients suffering from diabetes, which had contributed to their NAFLD, and the outcome of a subgroup meta‐analysis of 7 studies that only involved diabetic patients—both the NAFLD group and the control group—showed us that the presence of NAFLD, while slightly increasing the odds, was not significantly correlated with increase in CIMT (*p* value = 0.557; 1.089–0.955 confidence interval [CI] 95%; odds ratio [OR] = 1.020). This analysis can be observed in Figure 4.

We also assessed for the effect of the method of diagnosis (ultrasound vs. CT scan vs. biopsy) and noticed that there was no significant effect of method of diagnosis on the association between NAFLD and increase in CIMT (CT vs. biopsy: *p* = 0.72; ultrasound vs. biopsy: *p* = 0.22).

### 3.3 Association between NAFLD and atherosclerotic plaque in carotid arteries

**Figure 5:**
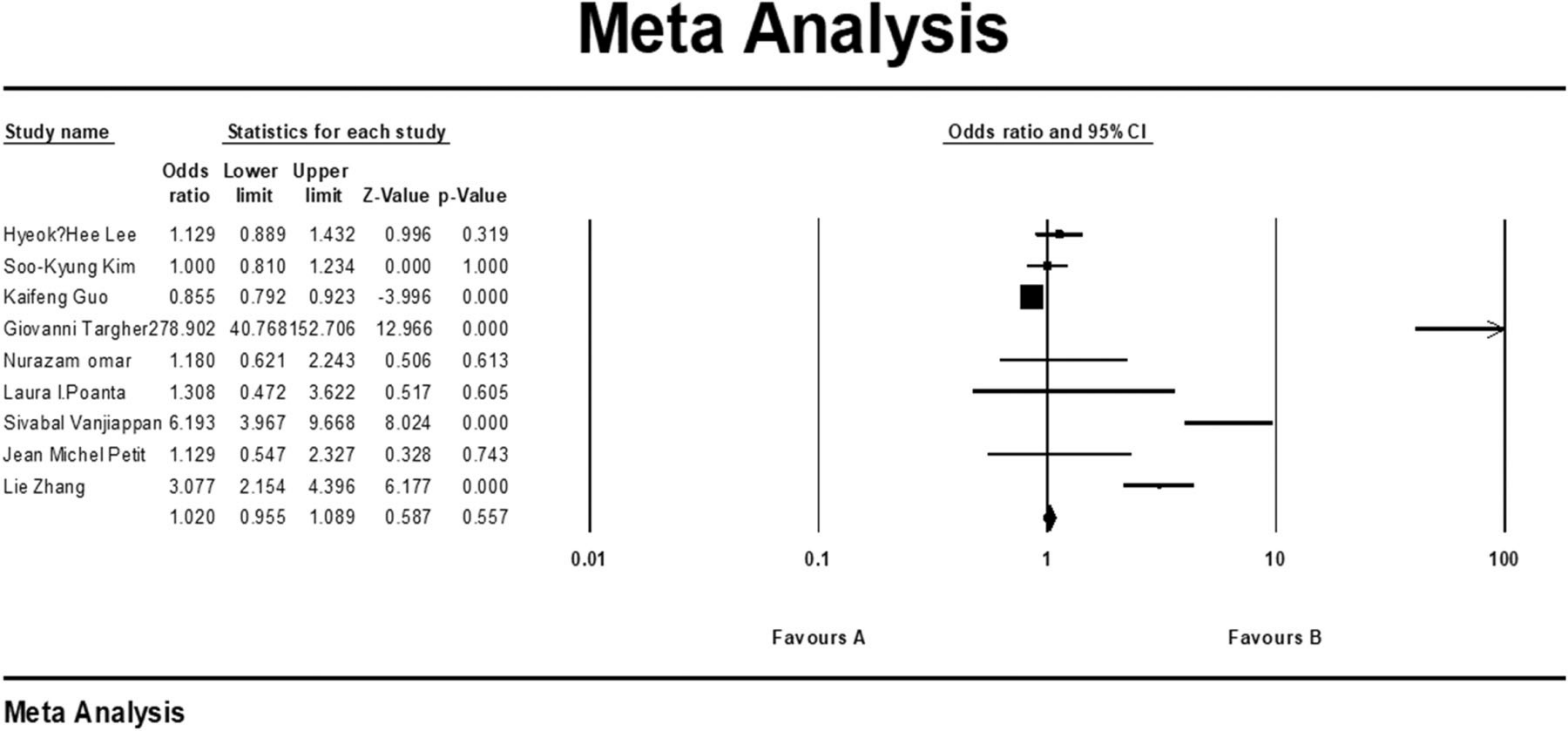
Results of a meta‐analysis of studies on the association of atherosclerotic plaque with NAFLD. CI, confidence interval; NAFLD, nonalcoholic fatty liver disease.

A meta‐analysis based on 17 separate studies revealed a significant association between the incidence of atherosclerotic plaques in the carotid arteries and the presence of NAFLD. They noted that in the patients with plaques, there was 1.35 times odds of having NAFLD versus those without plaques (*p* < 0.001, 1.28–1.43, 95% CI, OR = 1.356). The summary of this analysis can be viewed in Figure 5.

## 4 DISCUSSION

NAFLD has become a global public health issue since it is linked to metabolic risk factors such as obesity, diabetes mellitus, dyslipidemia, and metabolic syndrome, along with genetic, socioeconomic, and lifestyle components.^82^, ^83^, ^84^, ^85^, ^86^, ^87^, ^88^, ^89^, ^90^ Increased endothelial dysfunction,^91^, ^92^ ischemic heart disease,^93^ cardiovascular events,^93^, ^94^, ^95^ peripheral vascular disease, cardiovascular morbidity, and cardiovascular mortality are all medical issues, which may be caused by NAFLD.^87^, ^96^ Other research has shown an independent link between NAFLD and cardiovascular disease, regardless of other metabolic risk factors.^97^ This finding raises the possibility that NAFLD is a direct contributor to the pathogenesis of atherosclerosis rather than just a sign of cardiovascular disorder.^98^ Numerous pathogenic mechanisms, including elevated levels of oxidative stress brought on by steatosis‐stimulated fatty acid oxidation,^87^ systemic release of pro‐atherogenic molecules like tumor necrosis factor‐alpha, interleukin‐6, and oxidized low‐density lipoprotein cholesterol,^99^ elevated insulin resistance,^100^ and macrophage activation,^101^ have been proposed as potential causes for the acceleration of atherosclerosis and the rise in the prevalence of cardiovascular diseases in NAFLD patients. The atherogenic impact of liver inflammation is further confirmed by the fact that NASH (nonalcoholic steatohepatitis) patients had more atherosclerosis than steatosis patients.^102^, ^103^

In the present study, 59 observational studies were reviewed and the association between NAFLD and increased CIMT as well as prevalence of atherosclerotic plaque in the carotid artery (both subclinical indicators of atherosclerosis) was evaluated. In a pooled analysis of 59 studies, NAFLD was shown to be linked with a higher CIMT rate. In a pooled analysis of 59 studies, NAFLD was shown to be linked with higher CIMT. Of note, CIMT was 0.12 mm more (20.6%) in those with NAFLD than in controls (without NAFLD). Meanwhile, this value was about 18.7% in a meta‐analysis conducted by Madan et al. on 20 observational studies examining the influence of NAFLD on CIMT in adults,^104^ and 13% in a meta‐analysis performed by Sookoian et al. on 7 studies.^105^ In addition, a meta‐analysis of 17 studies revealed that NAFLD was related with a higher incidence of carotid plaque found by ultrasonography.^104^ In concordance to our findings, NAFLD was related with an elevated risk of carotid plaque (detected by ultrasonography) in the meta‐analysis of Madan et al.^104^ (of 13 studies). However, in a subgroup meta‐analysis of seven studies comparing diabetic individuals with NAFLD to diabetic patients without NAFLD, the presence of NAFLD was not significantly correlated with elevated CIMT rate in these patients. NAFLD seemed to be connected with an elevated risk of cardiovascular disease in both diabetic and nondiabetic individuals (T2DM).^106^, ^107^ Despite the fact that multiple research projects have shown that NAFLD is substantially correlated with higher CIMT in nondiabetic individuals, the association between fatty liver and atherosclerosis in patients with T2DM is less obvious, and there are contradictory findings across investigations.^16^, ^50^, ^69^, ^108^, ^109^ Targher et al. found that NAFLD measured by ultrasonography in T2DM patients on a restricted diet was related with an increased incidence of cardiovascular disease and CIMT.^109^ However, in agreement with our findings, Petit et al. did not demonstrate a link between NAFLD and elevated CIMT in T2DM patients.^69^ Similar to our investigation, Guo et al. also did not find correlation between NAFLD and elevated CIMT in T2DM patients in a Chinese hospitalized population controlling for multiple confounding factors.^25^ In contrast to our findings, Kim et al. observed that NAFLD is related to higher CIMT in individuals with T2DM but is impacted by insulin resistance.^16^ Guo et al., revealed that after controlling cardiovascular risk factors, there was an independent correlation between NAFLD and carotid and lower limb atherosclerotic plaques, which is indicative of the independent association between NAFLD and advanced atherosclerotic lesions in T2DM patients.^25^ The association between NAFLD and carotid atherosclerosis may be obscured by diabetes, which is regarded as one of the most significant risk factors for cardiovascular disease and the progression of atherosclerosis in the body. Other possible explanations for this contradiction include the techniques for detecting fatty liver (using ultrasound, CT, or magnetic resonance spectroscopy), ethnic disparities, and sample size.

Although increased CIMT has been shown to be associated with an increased risk of stroke,^93^ myocardial infarction,^93^, ^94^ and peripheral vascular disease,^93^ a recent meta‐analysis study suggested that carotid plaques may be a better predictor of cardiovascular risk than CIMT.^110^ In addition, comprehensive research revealed that carotid plaque area is a more accurate predictor of ischemic stroke in the first year than CIMT. Consequently, instead of CIMT, it is necessary to study the influence of NAFLD on the carotid plaque area in the next investigations.^111^

A recent meta‐analysis by Madan et al.^104^ of 28 studies revealed an increased risk of carotid atherosclerosis in adult and pediatric populations with NAFLD compared to groups without NAFLD. However, a number of other research have evaluated the connection between NAFLD and carotid disease.^11^, ^23^, ^24^, ^25^, ^26^, ^28^, ^30^, ^34^, ^41^, ^45^, ^48^, ^51^, ^52^, ^56^, ^58^, ^65^, ^73^ As a result, we did an updated meta‐analysis to incorporate new research done in Asia and Europe over the last years on the connection between NAFLD and carotid atherosclerosis through measurements of the CIMT in millimeters.

The constraints of any meta‐analysis research, by its nature, include its effect on the reviewed papers' texts, the risk of publication bias (publication bias), and the comprehensive search approach. To prevent this, we conducted the meta‐analysis using a comprehensive search approach and unambiguous inclusion and exclusion criteria. In addition, owing to the inclusion of observational studies in the analysis, unmeasured and underreported confounding factors and errors are possible. However, one of the most important strengths of our study was the focus on the amount of difference, along with percentage of difference, in the thickness of carotid intima‐media, instead of just describing the association or the odds of difference.

## 5 CONCLUSION

Through this systematic review and meta‐analysis, we concluded that NAFLD is correlated with an increase of 20.6% (0.12 mm) in CIMT. We also observed that NAFLD is correlated with an increase in atherosclerotic plaques.

## AUTHOR CONTRIBUTIONS

**Manouchehr Khoshbaten**: Conceptualization; data curation; formal analysis; writing—original draft. **Sepideh Hadi Maleki**: Data curation; formal analysis; writing—original draft. **Sara Hadad**: Data curation; formal analysis; validation; writing—original draft. **Amrit Baral**: Data curation; writing—original draft; writing—review & editing. **Ana Vitoria Rocha**: Data curation; investigation; writing—review & editing. **Laxmi Poudel**: Data curation; writing—original draft; writing—review & editing. **Alireza Abdshah**: Visualization; writing—original draft; writing—review & editing.

## CONFLICTS OF INTEREST STATEMENT

The authors declare no conflicts of interest.

## TRANSPARENCY STATEMENT

The lead author Alireza Abdshah affirms that this manuscript is an honest, accurate, and transparent account of the study being reported; that no important aspects of the study have been omitted; and that any discrepancies from the study as planned (and, if relevant, registered) have been explained.

## Data Availability

The data set of the extracted measurements, along with R codes for analysis and plotting, can be made available upon request.
